# 1370. Role of Clindamycin Versus Linezolid for Serious Group A Streptococcal Infections

**DOI:** 10.1093/ofid/ofab466.1562

**Published:** 2021-12-04

**Authors:** Emily Heil, Emily Heil, Sapna Basappa

**Affiliations:** 1 University of Maryland School of Pharmacy; University of Maryland Medical Center, Baltimore, MD; 2 University of Maryland School of Pharmacy, Baltimore, Maryland

## Abstract

**Background:**

*Streptococcus pyogenes* can cause severe illnesses such as toxic-shock syndrome and necrotizing fasciitis due to pyrogenic exotoxins. Clindamycin is added to penicillin for treatment of severe *S. pyogenes* infections as it is a bacterial protein synthesis inhibitor which reduces toxin production. However, clindamycin is associated with several adverse effects including *C. difficile* infection. Linezolid is a bacterial protein synthesis inhibitor that has been shown to provide excellent coverage of *S. pyogenes* including toxin inhibition in vitro, but clinical evidence is lacking. We compared outcomes of patients treated with linezolid versus clindamycin for serious *S. pyogenes* infections.

**Methods:**

This was a retrospective study of patients with necrotizing fasciitis or toxic shock syndrome caused by *S. pyogenes* admitted to the Shock Trauma Center at University of Maryland Medical Center treated with at least 48 hours of either clindamycin or linezolid. Data collected included Sequential Organ Failure Assessment (SOFA) and Laboratory Risk Indicator for Necrotizing Fasciitis (LRINEC) severity scores, time to resolution of infection, number of surgeries, *C. difficile* infection, other antibiotic associated adverse effects, and mortality. Associations between patient characteristics, antibiotic groups, and outcomes were analyzed using the chi-square test, Fisher’s exact test and t-test or Wilcoxon rank-sum test as appropriate (SAS v 9.4).

**Results:**

52 patients were included, 26 treated with clindamycin and 26 with linezolid. Most patients (85% clindamycin and 96.2% linezolid) were treated for necrotizing fasciitis. Baseline characteristics, including SOFA and LRINEC scores, were similar between the groups. There was no difference in mortality between patients treated with clindamycin versus linezolid (11.5% vs. 7.7%, p = 0.22), and resolution of infection was similar between the groups (92.3% vs. 88.5%, p = 1.0). There was no difference in adverse effects between the clindamycin and linezolid groups, including *C. difficile* infection (3.9% vs. 0% p = 1.0) and thrombocytopenia (30.8% vs. 42.3%, p = 0.4).

**Conclusion:**

Linezolid could be an alternate to clindamycin for the treatment of serious toxin producing *S. pyogenes* infections. Further prospective studies are needed.

**Disclosures:**

**Emily Heil, PharmD, MS, BCIDP**, Nothing to disclose

